# The Psychology of the Naturalist

**Published:** 1972-04

**Authors:** Philip Radford


### The Psychology of the Naturalist

**Philip Radford, M.B., Ch.B., D.C.H.**

Probably primitive man recognised the value of birds as food well before his mind became capable of understanding his jealousy of their powers of flight. That humans have long considered flying to be desirable is shown by early artists portraying angels, as spiritual super-humans, with functional wings; also, peoples such as the Assyrians carved bulls complete with wings, thus representing both strength and mobility. As well as hunting birds and mammals for food, early man must have looked for birds' nests. Clutches of eggs, in season, would have been prized as palatable food and in some tribal areas mystic significance may have been attributed to the markings and colours of the shells. Obviously, birds have always fascinated man. The Old Testament has many references to birds and so has early literature in general. In more recent times, Shakespeare's characters mentioned birds as omens, and included them in metaphoric and descriptive passages. This is not surprising as Shakespeare wrote in a community which lived close to nature, when even the largest towns, small by modern standards, were surrounded by woods, farms and open heaths.

Now that natural habitats, together with bird and mammal populations, have shrunk alarmingly it is to be expected that the increasing numbers of humans should demand some association with wild life. Man's complex brain has evolved for so long in close contact with nature that abrupt separation can lead to psychological disturbance. Hence, the modern interest in the study of birds and the multiplication of natural history societies could have been anticipated. To achieve a balance the energy of the human psyche, or in a conscious state, the ego, requires some interaction with the life of the moor, the forest and the estuary. Perhaps in the majority of cases such contact need only be superficial, but deeper involvement is required in other personality types. Today's ego needs the egg; the problem is to conserve the egg and to allow outlets for the necessary psychic drives. The ego itself is too destructive. Fundamentally man is selfish and unless the super-ego, the conscience, can instil understanding of the necessity of protecting the remaining natural environment, the countryside could disappear in its present form.

The development of man's higher cerebral functions has permitted aesthetic appreciation as well as curiosity. Thus, it is possible to admire the tones of the blue pigment of a male chalk-hill blue butterfly *Lysandra coridon* without studying the dead specimen in the laboratory. Again, the delicate form and shading of the flower of sheepsbit *Jasione montana,* can best be enjoyed in its setting on downs or coastal waste, but man also has the capability of dissecting the plant and taking pleasure in enquiring into the chemistry of its life. As another example, humans can appreciate the camouflage effect of the olive-green and buff mottlings of the egg of a sedge warbler *Acrocephalus schoenobaenus,* lying deep in its nest of dried grasses low in marsh vegetation; also, man can ask how the pigments became laid down in the shell of the egg and why some female sedge warblers lay eggs marked by black hair-lines. It is as though such eggs have had curved and irregular lines drawn on them in Indian ink by the use of a mapping pen. It can be understood how diffuse or dappled colours become incorporated in the calcium carbonate matrix during the formation of the egg by pigment cells of the bird's shell gland, yet how can a thin and discreet line be inscribed without being smudged? Similarly, man can admire the beauty of the blues and greens found on the bodies of common dor beetles *Geotrupes stercorarius* and can investigate whether the colours have biological significance. Then man may wonder if the common tiger beetle *Cicindela campestris,* with its bright green wing-cases, could catch, prey and breed effectively with wings of a duller hue?

### The Hunting Instinct

Nowadays, one of the most popular branches of natural history is ornithology, attracting a considerable number of competent amateur observers. Should such a bird-watcher, on a coastal marsh maybe, unexpectedly sight a rare wader through his binoculars, he gets a thrill of achievement which is probably very similar to that which a primitive hunter felt as his arrow sunk into the flank of a fleeing deer. Certainly hunting is a strong human instinct and man continues to hunt even though, in most cases, the prey is no longer essential as food; fishing, for example, remains as one of the most widely followed sports in Europe. Today, ornithologists hunt in many different ways. Some continue to trap birds, but instead of the catch being plucked for the pot its wing-formula is determined and it is weighed and a ring placed on the leg. Perhaps parasites are looked for amongst the feathers, as a kind of secondary hunting, then the bird is released: the bird-watcher has gratified his hunting instinct. Other ornithologists get pleasure from adding to their list of bird species seen and will travel great distances, like early hunters, to locate their quarry. Patience is necessary for any hunter: many naturalists are interested in bird behaviour and will wait for hours to observe a particular ceremony or mode of flight. Patience is also required by those who try to capture birds on photographic film, and surely a good bird photograph today has more real value than any dead bird. In the same way, the bird-watcher, who is often a bird-listener as well, uses his portable tape-recorder to capture the songs and calls of the birds he studies. Again, there is a sense of hunting fulfilment when a new song is recorded and, happily, this can be done with minimal disturbance of the bird and its surroundings.

After a successful hunt, man displays his trophies. Ornithologists of the past collected birds' eggs and skins, often exhibiting them with pride to their friends.

Fortunately, such days are almost over although the human wish to acquire is as strong now as at the time of the XIXth Century collectors. Now the naturalist must gloat over the reports of his ornithological investigations and his bird photographs or tape-recordings. One object a man might acquire today is a house and, basically, he defends it rather as a bird defends its territory in spring. If an ornithologist returns to find an intruder in his home he gets enraged, and should he discover that his favourite bird books and recordings have been stolen he will be still more enraged. A male song thrush *Turdus philomelos* chases other song thrushes from his breeding territory; similarly, man resents strangers entering his house uninvited.

**Aggressive Urge**

Acquisitiveness and hunting are associated closely with man's aggressive instinct. No doubt aggression is well modified and controlled by most naturalists, but at times it is a factor which is harmful to the animals they watch. For instance, a selfish photographer may cut back cover round a nest to get a better picture of the fledglings; again, bird-watchers have been known, sadly, to harass a tired and rare migrant to get a more detailed view of its plumage without any thought for its feeding requirements. Further to this, sometimes a naturalist develops an attitude of ambivalence towards birds; thus, he may shoot teal and mallard in the morning and delight in watching nuthatches searching for bark insects in the afternoon. Throughout history man has been aggressive to the extent of killing and wounding his fellows in the rival camp; it is not surprising, then, that occasionally his aggression against birds amounts to real cruelty. The boy who takes a pin and impales nestlings on a tree is, however, probably behaving no worse than a school bully who beats up a timid new boy.

Yet cruelty to birds and mammals need not be an inevitable component of man's aggressive urge; so much can be done to educate the young. Children are inquisitive and highly receptive as they develop; hence they can be taught. But if they are left entirely to their own devices even the more intelligent can become aimless, grubby and sometimes annoyingly destructive. Like birds, man cannot be reared without parental care but man's intelligence enables him to be so much more adaptable: he can invent and use machines and can live with success in a snow-bound city or as a nomad in a tropical desert. With such potential mental agility, most children are thrilled by watching birds but the interest can easily be lost; example by adults and subtle guidance will reinforce their enjoyment of nature and their respect for life in the wild. Probably all normal children are embryo naturalists but it is only by education that this can be realised.

It is likely that the association of juveniles or adults in gangs or herds increases aggressiveness. This may lead to another danger for birds because the larger a group of ornithologists becomes, the greater is their competitive spirit and zeal. But birds can hardly appreciate this enthusiasm; they cannot thrive when being chased by aggressive humans. Clearly, the mass watching of birds must be very carefully controlled and organised. The solitary or small group bird-watcher sees more of the birds and gets a deeper pleasure from the experience; certainly less disturbance is created for the birds he wants to protect. Of course, man is a sociable animal, in spite of his territorial and aggressive drives, and it is right that ornithologists should form societies for the exchange of bird information, to plan research projects and for educational purposes. It is of interest, however, that members of such clubs, or herds, elect their own officers and so establish a hierarchy, as an ornithological 'peck-order'. Humans are often classed as having an extrovert or introvert personality; usually it is the extrovert who is selected for office as he makes others aware of his capabilities. Bird-watching used to be regarded as a hobby for introverts, but modern ornithology has so many facets and poses so many questions that it has become a hobby of most personality types, as of all social classes. Probably it has always been so.

**Reproductive Urge**

One of the most powerful human instincts is the reproductive urge, and there seems to be no more popular subject for the press or television. Currently, it is the fashion to offer a sexual explanation for many human difficulties. For this reason, it may be refreshing to think of ornithology as having little connection with sex or, at any rate, human sex. Yet, apart from birds' reproductive biology, it is possible that birds have sexual symbolism for some humans, in a psychological sense. Birds have long, probing bills and some sing at night in romantic settings; others enter deep nest-cavities; these facts could have sexual linkage in the subconscious mind. It could be significant that birds often appear in the dreams and fantasies of disordered persons although, for that matter, interested normal humans often have frequent bird dreams. On a more realistic level, human pair-bonds have been known to start in the natural history society; there is truth in the proverb: 'birds of a feather flock together'. But whether they are paired or not, bird-watchers gratify some of their innate parental drives when they see fledglings reared to the free-flying stage, and especially those from a nest in their own gardens, and perhaps from a nest-box constructed and sited by their own hands.

While sexual and aggressive motives are fundamental to man's life, he has evolved to something higher. Human civilisation is dependent on man's appreciation of beauty and a wish to create objects of beauty. Many people take up bird-watching because of their aesthetic sense while others combine this with the added interest of scientific investigation. As was noted previously, man's brain is so versatile that it is quite possible to enjoy the appeal of a charm of goldfinches *Carduelis carduelis* taking seed from a thistle patch and to get satisfaction from the statistical and mathematical analysis of the numbers involved. Those who have artistic ability must be excited by capturing a bird's movement and vitality of colour in a painting; it seems obvious that no bird artist could be successful unless he had watched birds in their natural habitat. And it is in birds' habitats that the ornithologist can get such mental stimulation. Considering man's basic instincts it is unnatural that families should live in multi-storey blocks of flats in crowded, noisy and smelly towns. To escape to the country, whether in storm or sunshine, and to watch birds feeding and calling along a moorland stream unaccompanied by the roar of motor traffic, gives the city dweller a feeling of contentment not easily obtained in the world today.

Natural history is so fascinating a subject that a psychological fixation on it is quite understandable. Early man lived and hunted in forests and on open plains; the brain of modern man cannot lose all con-ection with the past. Some contact with the countryside is necessary if the nervous tensions and strains of busy urban life are to be avoided. What better means is there of doing this than by becoming a bird-watcher, an entomologist or a botanist?

